# Parafoveal syntactic processing from word N + 2 during reading: the case of gender-specific German articles

**DOI:** 10.1007/s00426-023-01833-9

**Published:** 2023-05-20

**Authors:** Laura Schwalm, Ralph Radach

**Affiliations:** University of Wuppertal, Max-Horkheimer-Str. 20, 42119 Wuppertal, Germany

## Abstract

Previous research has suggested that some syntactic information such as word class can be processed parafoveally during reading. However, it is still unclear to what extent early syntactic cueing within noun phrases can facilitate word processing during dynamic reading. Two experiments (total *N* = 72) were designed to address this question using a gaze-contingent boundary change paradigm to manipulate the syntactic fit within a nominal phrase. Either the article (Experiment 1) or the noun (Experiment 2) was manipulated in the parafovea, resulting in a syntactic mismatch, depending on the condition. Results indicated a substantial elevation of viewing times on both parts of the noun phrase when conflicting syntactic information had been present in the parafovea. In Experiment 1, the article was also fixated more often in the syntactic mismatch condition. These results provide direct evidence of parafoveal syntactic processing. Based on the early time-course of this effect, it can be concluded that grammatical gender is used to generate constraints for the processing of upcoming nouns. To our knowledge, these results also provide the first evidence that syntactic information can be extracted from a parafoveal word N + 2.

## Introduction

For over 4 decades, substantial research work has been devoted to eye movement analyses of real time processing of words and sentences during reading (Radach & Kennedy, 2013; Rayner, 2009). Within this field, a central line of research has focused on the spatially distributed nature of linguistic processing. There is a rich literature on parafoveal processing of words within sentences, but within this literature, very little attention has been paid to syntactic aspects of reading so far. This is somewhat surprising, as the processing of syntax is one of the central topics of psycholinguistic research in general, including quite a bit of eye movement work (see Clifton et al., 2007, for a seminal review on eye movement research on the sentence level).

A central theoretical question is whether syntactic properties words within the context of a sentence are processed prelexically at an early stage or only postlexically. Answers given to this question are intimately related to the wider issue of modularity vs. interactivity in language processing. Proponents of interactive approaches suggest that the interaction of semantics and syntax pre-activates lexical candidates for the following word (Friederici & Jacobsen, 1999). Context (both semantic and syntactic) is thought to reduce the search effort for matching items in the lexicon (Bates et al., 1996). Accordingly, information about a word category or grammatical gender would lead to the pre-activation of a lexical subgroup, facilitating the processing of congruent and inhibiting incongruent words.

An alternative theoretical position is taken in modular models of lexical access (see, e.g., Tanenhaus & Lucas, 1987, for a detailed discussion). Here, the language processing system is assumed to consist of functionally autonomous modules. For each particular module, the relation between input and output is seen as independent of the information processed by other modules. This view, referred to as the autonomy hypothesis, makes two predictions with respect to lexical processing: (1) the information acquired at each stage is invariant with respect to different processing contexts and (2) the rate at which this information becomes available is also not dependent on context. Modules are assumed to communicate only at the ends of input and output, so that the output of one module serves as input to the subsequent one. Consequently, the internal processes of one module do not have access to the process status of other. With respect to syntax in written language, this would mean that it is processed postlexically and has no influence on the availability or pre-activation of words as reading progresses through a sentence. One theoretical argument for this view is that that pre-activation of all matching words available in the lexicon would involve far too much computational effort (Tanenhaus & Lucas, 1987).

Different hypotheses with regard to the processing of syntactic information during reading can be derived on the basis of the two classes of models. Interactive models suggest early processing of syntactic information and therefore predict both inhibitory effects in the presence of incongruent syntax and facilitatory or pre-activating effects in the presence of congruent syntax. The modular models, on the other hand, postulate post-lexical processing of syntax and therefore predict only inhibitory, but no facilitatory (i.e., priming) effects.

Behavioral and neurophysiological studies in which subjects were exposed to semantic and syntactic errors provide data relevant to the issue. Commonly observed event-related potentials (ERPs) during the reading of rule-violating sentences are the N400, which is observed in the processing of mismatching semantic information (Kutas & Federmeier, 2007) and the P600, which is related to syntactic information processing (Friederici, 1999; Hagoort et al., 1993; Hagoort, 2003, for a review, see Molinaro et al., 2011). Early left anterior negativity (< 200 ms) is observed during the processing of an incongruent sentence structure, word category (Friederici, 2002) or genus incongruence (Guajardo & Wicha, 2014). To make more specific statements about the temporal course of syntax processing, Deutsch and Bentin (2001) conducted experiments on syntactic priming and concluded on the basis of ERPs that syntactic information of words is processed at an early stage, even if interaction with semantics only occurs postlexically.

Only a few recent studies have examined early processing of syntactic information during reading using eye movement methodology. Brothers and Traxler (2016) tested the assumption that each word within a sentence generates an expectation that acts to constrain the syntactic category of subsequent words. According to these authors, early processing of word class constraint may take place in an anticipatory fashion, therefore facilitating lexical access. They hypothesized that word class congruency effects should become manifest on early eye-tracking measures such as the likelihood for fixating a word, usually referred to as skipping rate (1—fixation likelihood). To test their assumptions, they conducted three experiments in which syntactic congruence of verbs or nouns was manipulated using the boundary technique of saccade contingent display changes (see our method section below for a description). As one key result, they observed a lower fixation frequency (higher skipping rate) for syntactically valid (*The admiral would not confess…*) compared to invalid previews (*The admiral would not surgeon…*). It is well established that word repetition facilitates early lexical processing and leads to higher skip rates (Traxler et al., 2000). In a third condition (*the admiral would not admiral…*), this effect was shown to be inferior to syntactic fit in Brothers and Traxler (2016). The authors take this finding to imply that word class constraints may take precedence over lexical repetition effects. Overall, they conclude that their data are most consistent with an anticipatory account, in which readers preactivate syntactic constraints on upcoming words and immediately use these constraints to facilitate early stages of lexical identification.

Similar findings were obtained in a study by Snell et al. (2017). In one of their experiments using the boundary method, they presented sentences in which the target in the parafovea was either identical, congruent or incongruent with respect to part of speech. They reported higher fixation probabilities and longer first-pass fixation viewing times on the target when it was incongruent. These authors also employed a flanker paradigm in which they presented target words in the fovea that were combined with words on the left and right, resulting in syntactically congruent vs. incongruent combinations. In this task, response times and error rates were substantially lower in the congruent flanker condition. This was taken to indicate that lexical information may be gathered and integrated in parallel across multiple words, allowing for detection of a syntactic congruence vs. incongruence.

According to Veldre and Andrews (2018), however, the findings of the two studies discussed above might be attributed to plausibility rather than syntactic effects. They conducted two further experiments in which syntactic fit and plausibility were manipulated separately. Both experiments showed unique significant benefits for syntactically correct previews for early oculomotor parameters such as first fixation duration but also gaze duration and go-past time. Significant effects for fixation probability were found in one of the two experiments. The authors conclude that syntactic word class information is indeed processed relatively early during the course of sentence reading.

Cutter et al. (2020) conducted a study to investigate fixation probabilities while reading contextually predictable or unpredictable words in both syntactically legal and illegal positions. They observed that unpredictable words in a syntactically legal sentence context were associated with higher fixation probabilities, but there were no clear effects for the illegal condition. These results suggest that syntactic information is processed parafoveally and may be more influential than predictability information, or at least that the two types of information interact.

In addition to word class, a second important type of syntactic information is grammatical gender. In many languages like Spanish, French, and German, explicit grammatical gender is a ubiquitous part of syntax. In German, a minimal noun phrase consists of an article and a noun with explicitly marked gender correspondence. For example, “the house”, “the tree”, and “the bench” are expressed in German as “das Haus” (neuter), “der Baum” (male), and “die Bank” (female). The article specifies the noun in terms of singling it out as one specific object from the set of possible objects that can be denoted by a noun—e.g., a chair from the set of all chairs (Schmuck, 2020; Vater, 1984). Articles as part of a noun phrase provide information on case, number, and gender of the corresponding noun. Collectively, these features are referred to as phi-features (Adger & Harbour, 2008).

Some evidence for the use of gender correspondence comes from gender priming in word recognition tasks (Friederici & Jacobsen, 1999). In experiments where a prime provides a useful vs. misleading syntactic cue to the target, robust effects of syntactic inhibition were found, whereas effects of facilitation appear inconsistent. Facilitation effects occur especially when auditory sentence material is involved and more so in Lexical Decision Tasks compared to Naming Tasks. In addition, the effect appears more frequently in phonologically transparent languages, since in these, it is possible to infer sound properties of the noun on the basis of grammatical gender (see Friederici & Jacobsen, 1999, for a review).

We are aware of the fact that results like this can only be transferred to continuous reading with caution. According to Kuperman et al. (2013), only a small proportion of the variance in viewing times in natural reading can be explained by reaction times in the lexical decision task. Similar doubt may be in place vis-à-vis results of studies in which overt violations of gender matching are presented during the reading of sentences, as was done in electroencephalogram (EEG) studies, showing N400 effects of syntactic mismatch (e.g., Gunter et al., 2000). Effects in such studies occur in response to massive disruption of sentence processing and might not be fully representative of natural reading.

Our work is the first to study gender effects within noun phrases during normal reading for meaning. Our intention was to examine whether gender information provided by the article in a phrase like “die Musik” (the music, _female_) or “der Klang” (the sound, _male_) is utilized when this information becomes available in the parafovea during the reading of sentences. The alternative would be that fluent readers ignore this syntactic cue and focus processing on the noun as soon as its letters become parafoveally visible. The example of English provides ample evidence that the processing of noun phrases can proceed smoothly without the presence of any gender-specific cues.

We used the boundary method of saccade contingent display changes (Schotter et al., 2012) to provide either a gender match or a mismatch between article and noun as long as the eyes were fixating positions to the left of an invisible boundary. The boundary was located immediately to the right of a verb preceding the noun phrase. When the eyes crossed this invisible line, any mismatching parafoveal preview was restored, so that the noun phrase appeared completely normal as soon as it was fixated. The gender match between the first part of the sentence (up to the verb) and the subsequent article was always preserved, so that both versions of the article formed a grammatically and semantically suitable continuation of the sentence (see Appendix for all materials). Therefore, any effect in terms of increased viewing times would be based entirely on the parafoveal processing of the syntactic relationship between article and noun. To examine both parts of the nominal phrase, the article and the noun were manipulated in separate experiments. We suspected that early utilization of gender information might only take place when concurrent lexical processing allows for sufficient cognitive resources. Therefore, the frequency of the noun within the critical phrase was manipulated as a second factor, based on the seminal work of Inhoff and Rayner (1986) who first showed that frequent words are more effectively processed in the parafovea.

## Experiment 1

In the experiment, participants were asked to read a series of sentences in which a sequence of verb, article and noun was placed at a central position within the sentence. The sentences were presented in one of four conditions, with either a preview of a high vs. low-frequency noun, combined with a fitting or non-fitting article. In the fitting condition, the article and the noun shared the same grammatical gender, resulting in a grammatically correct sentence. In contrast, in the non-fitting condition, an incorrect article was presented before the noun, resulting in a grammatically incorrect noun phrase in the parafovea (e.g., the noun is feminine and the article is masculine). An invisible boundary was placed at the left edge of the empty space before the article, so that when the eye had crossed the boundary, the correct article was displayed in foveal vision.

Importantly, the article was always consistent with the preceding verb, so that a parafoveal syntactic mismatch could only be noticed after the noun had been processed. If grammatical gender information can already be processed in the parafovea, longer viewing times and higher fixation probabilities on the article are to be expected. However, as the syntactic fit can only be verified after the noun has been processed (N + 2), higher viewing times should also occur on the noun in the case of a syntactically mismatched preview. Since the processing effort for high-frequency words in the parafovea is substantially lower (Inhoff & Rayner, 1986), this effect should be more pronounced in high-frequency nouns.

### Method

#### Participants

36 subjects took part in the experiment. 33 of these were undergraduates at the University of Wuppertal. All were native German speakers with normal or corrected-to-normal vision and were unaware of the purpose of the experiment. Participants received partial course credit as compensation.

Power analyses were conducted in R (Version 3.5.2; R Core Team, 2017) using the mixedpower package (Version 0.1.0; Kumle, et al., 2021) following approaches recommended by Kumle et al. (2021) for estimating power for linear mixed models. The sample size was selected to ensure that simulations demonstrated a detection power of over 80% for even small effect sizes.

#### Materials and design

Stimuli consisted of 96 single-line sentences, 77–83 letters in length. At a central position within the sentence, a common sequence of verb, article, and noun was presented (see Appendix for a list of all the sentences used). The noun was five-to-six letters long and either high or low in frequency. High-frequency words were chosen as having a frequency count of at least 30 occurrences per million (opm) and low-frequency words had a frequency count of less than 10 per million based on the speech norms in the SUBTLEX corpus (Brysbaert et al., 2012). The mean word frequency for the low-frequency group was 3.8 opm with a standard deviation of 2.87 opm and a range between 0.04 and 9.76 opm. For the high-frequency condition, the mean was 93 opm with a standard deviation of 119.49 opm and the values ranked between 10.35 and 569.96 opm.

**Fig. 1:**
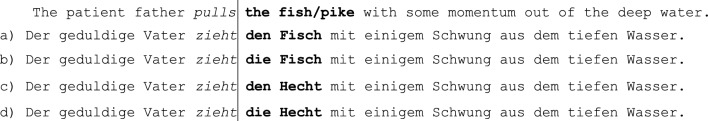
An example sentence in each of the four conditions in Experiment 1, along with a literal translation into English 1: **a** high frequency, identical, **b** high frequency, syntactic mismatch, **c** low frequency, identical, **d** low frequency, syntactic mismatch

The boundary paradigm was used to manipulate parafoveal preview of the article. Four conditions were created: (1) Identical, HF: identical article, high-frequency noun, (2) Identical, LF: identical article, low-frequency noun (3) Mismatch, HF: syntactically mismatching article, high-frequency noun (4) Mismatch, LF: syntactically mismatching article, low-frequency noun. A sample of sentence pairs is shown in Fig. 1. Sentences appeared in all conditions across four counterbalanced lists.

##### Stimulus validation

A separate set of 30 subjects participated in a cloze norming task to evaluate the predictability of the noun. This norming task indicated that sentence contexts were neutral, with on average the low-frequency noun only being produced in 0.94% (SD = 3.9%) of the time and the high-frequency noun in 1.7% (SD = 3.8) of the time. There was no difference in the predictability of the high- and low-frequency nouns, *F*(1, 190) = 2.22, *p* = 0.138.

#### Apparatus

Eye movements were recorded using an SR Research Eyelink 2 k eye tracker running at a sampling rate of 2,000 Hz. Sentences appeared in single lines in black monospaced font on a light gray background. Participants were seated approximately 70 cm away from a 21-inch CRT monitor with a screen resolution of 1,680 × 1,050 pixels and a refresh rate of 120 Hz. A chin and forehead rest were used to minimize movements of the head. At this distance three characters subtend approximately 1° of visual angle.

#### Procedure

Participants were asked to read each sentence at their normal pace to understand its basic meaning. At random intervals (on average after four sentences), a comprehension question appeared that required a verbal response. Comprehension questions targeted sematic relations within sentences, ensuring reading for meaning (Radach et al., 2008). The experiment started with a three-point calibration procedure followed by four practice trials. The 96 experimental sentences were then presented intermixed with 95 filler sentences, which varied in their grammatical structure. At the beginning of each trial, the subjects were presented with a fixation cross in the same position as the first letter of the sentence. Tracker accuracy was monitored throughout the experiment, and re-calibrations were performed every five trials, after each comprehension question or when calibration error exceeded 0.3 degrees of visual angle. When the current trial was completed, the participant pressed a button to advance to the next sentence.

### Results

Fixations below 70 ms or above 600 ms were eliminated (0.8% of total fixations). Gaze durations above 1,000 ms (0.7% of trials), and go-past times above 2000 ms were also excluded (0.5% of trials). Trials in which the subject blinked immediately before or after fixation were eliminated (3.05% of trials). These exclusions left 3272 trials (96.91% of the data) available for analysis. On average, 96.3% of comprehension questions were answered correctly, indicating successful reading for meaning.

The following oculomotor measures were analyzed for the article and the target noun (see Inhoff & Radach, 1998, for a general discussion of eye movement measures): the *fixation probability* (probability of a word to be fixated during first-pass reading); *first fixation duration* (duration of the first fixation on the target word), *gaze duration* (sum of all fixations on the target word before moving to another word), *go-past duration* (sum of all fixations made from the moment the eyes land on the target word until the first fixation to the right of the target word*)*, and the *probability for a regression out* (probability of the text fixation to land on a word left to the current fixation). Average subject means for the two mask conditions on each of these measures are presented in Table 1.

**Table 1:** Mean (and Standard Deviation) eye movement measures (based on means per participant and cell of the design) for Target Words (Article and Noun) across Preview Conditions in Experiment 1

	Article		Noun	
	Identical	Mismatch	Identical	Mismatch
Fixation probability (%)	61.5 (16.8)	70.8 (18.1)	86.5 (9)	89 (7.1)
First fixation duration (ms)	208 (30)	222 (33)	209 (24)	219 (27)
Gaze duration (ms)	216 (34)	242 (49)	236 (35)	245 (39)
Go-past duration (ms)	264 (61)	312 (95)	317 (74)	384 (93)
Regression out (%)	17.7 (14.6)	18.2 (13.9)	21.4 (18.2)	29.3 (18.9)

Data were analyzed using (generalized) linear mixes models (LMM) with the lme4 package (Version 1.1-18-1; Bates et al., 2015) in R (Version 3.5.2; R Core Team, 2017). Unless otherwise noted, log-transformed data yielded the same pattern of significance as the analysis based on the raw data. To ease communication, we therefore report analyses of the untransformed data. The random effect structure of the models included slopes for all the fixed effects across subjects and items, including interactions (Barr et al., 2013), and were only trimmed down if the model did not converge. Absolute *t* values equal to or greater than 1.96 were interpreted as significant, given the number of observations, the *t* statistic in LMMs approximates the *z* statistic.

For all analyses, we included as fixed factors mask condition and noun word frequency. High-frequency nouns were coded as “1” and low-frequency nouns were coded as “2”. Originally, prior gaze duration and launch distance relative to the target word had also been included in the model, but both showed no significant interactions with the mask condition and were therefore removed from the models. The fixed effect estimates for the article are shown in Table 2 and for the noun in Table 3.

**Table 2:** Results of the (generalized) linear mixed-effects models for fixation duration and probability measures for the article in Experiment 1

Measure	Fixed effect	*b*	SE	*t/z*
Fixation probability (%)	Intercept	**0.44**	**0.14**	**3.19**
	Noun Frequency effect	0.19	0.11	1.74
	Syntactic validity preview effect	**0.52**	**0.3**	**4.17**
	Noun Frequency effect t: Syntactic validity preview effect	− 0.9	0.16	− 0.6
First fixation duration (ms)	Intercept	**209.83**	**5.57**	**37.66**
	Noun Frequency effect	− 2.71	5.14	− 0.52
	Syntactic validity preview effect	**13.77**	**5.2**	**2.65**
	Noun Frequency effect: Syntactic validity preview effect	0.7	7	0.1
Gaze duration (ms)	Intercept	**214.6**	**7.62**	**28.16**
	Noun Frequency effect	2.85	6.64	0.43
	Syntactic validity preview effect	**29.86**	**6.45**	**4.63**
	Noun Frequency effect: Syntactic validity preview effect	− 3.56	9.04	− 0.39
Go-past duration (ms)	Intercept	**259.65**	**14.91**	**17.42**
	Noun Frequency effect	7.7	13.74	0.56
	Syntactic validity preview effect	**63.39**	**13.33**	**4.75**
	Noun Frequency effect: Syntactic validity preview effect	− 22.56	18.69	− 1.21
Regression out (%)	Intercept	**1.87**	**0.2**	**9.62**
	Noun Frequency effect	− 0.14	0.18	− 0.76
	Syntactic validity preview effect	− 0.14	0.2	− 0.71
	Noun Frequency effect: Syntactic validity preview effect	0.2	0.24	0.82

Significant effects are indicated in bold

**Table 3:** Results of the (generalized) linear mixed-effects models for fixation duration and probability measures for the noun in Experiment 1

Measure	Fixed effect	*b*	SE	*t/z*
Fixation probability (%)	Intercept	**1.84**	**0.15**	**12.14**
	Noun Frequency effect	**0.5**	**0.15**	**3.28**
	Syntactic validity preview effect	**0.3**	**0.14**	**2.21**
	Noun Frequency effect: Syntactic validity preview effect	− 0.17	0.22	− 0.78
First fixation duration (ms)	Intercept	**203.92**	**4.24**	**58.86**
	Noun Frequency effect	**9.82**	**3.35**	**2.93**
	Syntactic validity preview effect	**8.96**	**3.31**	**2.71**
	Noun Frequency effect: Syntactic validity preview effect	0.93	4.65	0.2
Gaze duration (ms)	Intercept	**224.2**	**6.4**	**35.02**
	Noun Frequency effect	**22.03**	**4.95**	**4.45**
	Syntactic validity preview effect	**11.08**	**4.93**	**2.25**
	Noun Frequency effect: Syntactic validity preview effect	− 3.04	6.94	− 0.44
Go-past duration (ms)	Intercept	**292.79**	**14.01**	**20.91**
	Noun Frequency effect	**48.99**	**13.52**	**3.62**
	Syntactic validity preview effect	**51.18**	**14.89**	**3.44**
	Noun Frequency effect: Syntactic validity preview effect	30.04	18.96	1.58
Regression out (%)	Intercept	**1.87**	**0.22**	**8.31**
	Noun Frequency effect	− **0.37**	**0.16**	− **2.26**
	Syntactic validity preview effect	− **0.68**	**0.16**	− **4.25**
	Noun Frequency effect: Syntactic validity preview effect	0.16	0.2	0.81

Significant effects are indicated in bold

#### Article viewing times

**Fig. 2:**
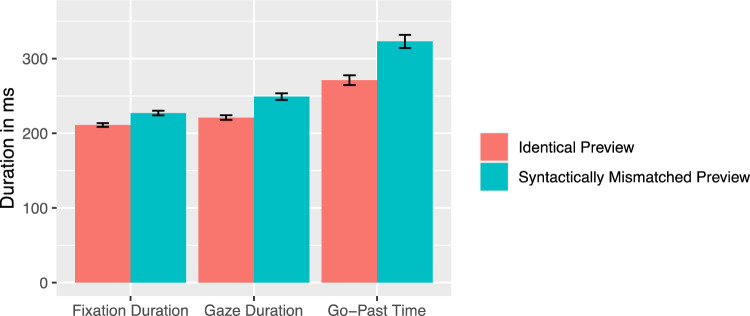
Viewing times on the article as a function of preview condition (Experiment 1). Note. Error bars represent ± 1 standard error of the mean, calculated within-subjects

On the article, fixation probability was higher for the syntactically mismatching mask [*z* = 4.17]. There was a significant syntactical preview effect across all three viewing time measures, as readers spent less time fixating the article when the preview was syntactically matching the noun (all |*t*|s > 2.65; see Fig. 2). The mask condition had no influence on the probability of an outgoing regressive saccade [*z* = − 0.71].

#### Noun viewing times

**Fig. 3:**
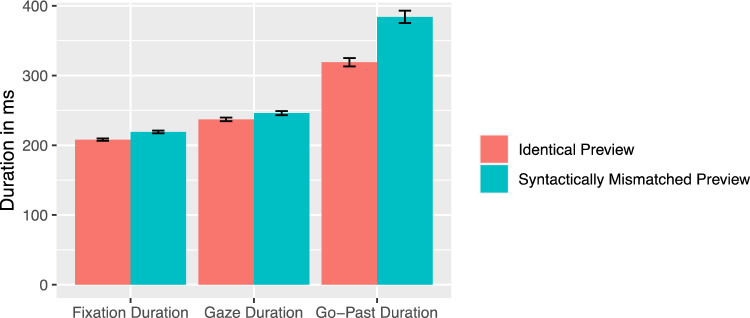
Viewing times on the noun as a function of preview condition (Experiment 1). Error bars represent ± 1 standard error of the mean, calculated within-subjects

**Fig. 4:**
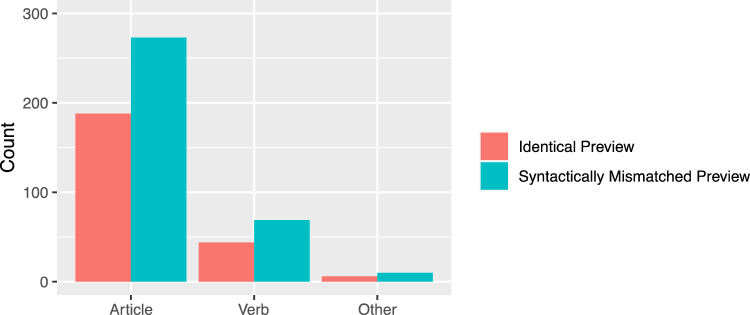
Regressions out of the noun as a function of preview condition and target word (Experiment 1)

On the noun, the fixation probability was higher for the syntactically mismatching mask [*z* = 2.21]. In addition, all viewing times measures showed a reliable effect of the mask condition. First fixation duration, gaze duration, and go-past duration were substantially increased for the syntactically mismatching mask of the article (all |*t|*s > 2.25, see Fig. 3). The analysis of regressions out revealed that readers were more likely to start a regression out of the noun, if the article mask was syntactically incorrect [*z* = − 4.25]. Further analysis of the regression target revealed that with 77.8% most of the regressions landed on the article, 19.8% on the verb, and 2.3% on other words at the beginning of the current sentence (see Fig. 4 for absolute values). Numerically, the article appeared to be viewed again most frequently when a syntactically inappropriate mask was presented. However, there was no significant difference between the mask conditions for the regression target [*b* = 0.03, SE = 0.23, *z* = 0.12].

### Discussion

In Experiment 1, subjects read sentences for comprehension that included a parafoveally matched or mismatched nominal phrase. The aim of the study was to determine whether and at what stage gender information within phrases is utilized during reading in German.

Our results indicate a reliable effect of gender marking. The fixation probability of the article decreased when an identical word mask was presented. Accordingly, when a gender mismatch was presented in the parafovea the article was skipped less frequently. First fixation duration, gaze duration, and go-past time were also increased when a syntactic mismatch was presented. Equally pronounced was the effect of parafoveal gender mismatch on the associated noun. A non-matching article in the parafovea led again to longer viewing times for all parameters included. Based on these results, it can be concluded that the entire nominal phrase was processed in the parafovea, enabling the detection of a gender mismatch. Particularly the clear effects on the noun, even though this word was not manipulated between parafoveal and foveal viewing, provide evidence for a joint processing of the nominal phrase in terms of a “word group” (Radach, 1996; see Drieghe et al., 2008 for studies on word group effects in English).

Interestingly, we found no significant interaction involving the preview condition. The gender preview effect appears to be independent of noun frequency, long vs. short prior gaze duration, and far vs. near launch distance. It might be the case that the replacement of the extremely frequent article has directed attention to its location, as evident in the large number of regressions back to this position. This may have obscured an effect of noun frequency. The missing interaction with noun frequency may also raise the question if the longer viewing times on the noun were the result of a spillover effect rather than evidence for parafoveal processing of the nominal phrase (e.g., Findelsberger et al., 2019; Schroyens et al., 1999). Furthermore, a shortcoming of Experiment 1 was the lack of an appropriate baseline condition. In the two conditions with gaze-contingent display changes, the changed stimuli were both syntactically and orthographically different (e.g., “den Fisch” vs. “die Fisch”). It could therefore be argued that the longer viewing times are to some extent a result of the orthographic changes.

To exclude the possibility that a spillover or an orthographic preview effect might be the source for the observed longer viewing times on the noun, this word itself was masked in a second experiment. In contrast to Experiment 1, word N + 2 was manipulated instead of word N + 1, allowing for a neutral baseline (cf. Veldre & Andrews, 2018). In addition to the no change and the syntactically invalid mask, a syntactically valid, gender matching mask was presented as a third condition in the parafovea.

## Experiment 2

### Method

#### Participants

For Experiment 2, the sample comprised 36 participants with a mean age of 26.4 years. Most of them (*n* = 30) were undergraduate students from the University of Wuppertal. None of the participants had completed Experiment 1 or any of the validation studies. All were native German speakers with normal or corrected-to-normal vision and were naïve to the purpose of the experiment. Participants received partial course credit as compensation. Power analysis was performed using the same procedure as in Experiment 1.

#### Material and design

The stimuli were 84 single-line sentences, 77–83 letters in length. In a central position within the sentence, a sequence of verb, article, and noun was presented (see Appendix for a list of all sentences used). The verb and the noun were both five-to-six letters long and high in frequency with frequency counts of at least 30 occurrences per million, according to the part-of-speech norms in the SUBTLEX corpus (Brysbaert et al., 2012). There was no difference in lexical frequency between the three preview conditions (*F*(2) = 0.35, *p* = 0.7).

**Fig. 5:**
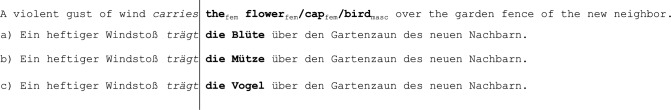
Example sentence in each of the three conditions from Experiment 2: **a** identical, **b** syntactic match, and **c** syntactic mismatch

This time the boundary paradigm was used to manipulate the parafoveal preview of the noun. The invisible boundary was placed right behind the verb resulting in an N + 2 manipulation. Three different preview conditions were created: (1) identical noun, (2) syntactically matching noun, and (3) syntactically mismatching noun. Each noun was presented in a sentence context that was designed to be neutral and not predict one of the three preview nouns (all cloze scores < 0.05). A sample of sentence pairs is shown in Fig. 5. All sentences appeared in all conditions across four counterbalanced lists.

#### Apparatus

The apparatus and display parameters were identical to Experiment 1.

#### Procedure

The procedure was identical to Experiment 1.

### Results

Data were trimmed identically to Experiment 1. Very short or long fixation durations (1.3%), gaze durations (0.8%), and go-past durations (0.9%) were excluded. In addition, trials in which the subject blinked immediately before or after fixation were eliminated (2.53% of trials). These exclusions left 2,620 trials (95.53% of the data) available for analysis. Participants answered an average of 94.36% of the comprehension questions correctly.

The same *eye movement measures* were analyzed as in Experiment 1. Mean values for the different preview conditions are presented in Table 4. Contrasts were set to compare the differences between (1) the identical preview with the syntactically matching preview and (2) the syntactically matching with the non-matching preview. Through this procedure, evidence can be obtained on whether there are differences between a purely orthographic and an additional syntactic change, therefore providing an adequate control condition. In Tables 5 and 6, the (G)LMM estimates for coefficients, standard errors, and *t*/*z* values for the fixed effects are reported.

**Table 4:** Mean (and standard deviation) eye movement measures for the target words (article and noun) across preview conditions in Experiment 2

Measure	Article			Noun		
	Preview condition			Preview condition		
	Identical	Syntactical match	Syntactical mismatch	Identical	Syntactical match	Syntactical mismatch
Fixation probability (%)	68.3 (20)	66.4 (20.4)	70.1 (20.4)	86.6 (11.6)	91.6 (7.6)	90.1 (9.6)
First fixation duration (ms)	217 (36)	226 (34)	230 (41)	220 (28)	237 (30)	238 (32)
Gaze duration (ms)	230 (47)	238 (45)	245 (51)	240 (40)	267 (40)	268 (47)
Go-past time (ms)	306 (87)	322 (99)	332 (99)	336 (86)	393 (100)	404 (118)
Regression out (%)	18.1 (11.1)	21.1 (15.8)	18.9 (13.4)	25.1 (23.5)	28.7 (21.6)	31.1 (25.6)

**Table 5:** Results of the (generalized) linear mixed-effects models for fixation duration and probability measures for the article in Experiment 2

Measure	Fixed effect	*b*	SE	*t/z*
Fixation probability (%)	Intercept	**0.92**	**0.17**	**5.52**
	Orthographic preview effect	− 0.01	0.07	− 0.02
	Syntactic validity preview effect	0.12	0.07	1.64
First fixation duration (ms)	Intercept	**227.85**	**5.6**	**40.67**
	Orthographic preview effect	− **6.64**	**2.89**	− **2.3**
	Syntactic validity preview effect	4.2	3.24	1.3
Gaze duration (ms)	Intercept	**234.68**	**8.23**	**28.52**
	Orthographic preview effect	6.2	5.82	1.07
	Syntactic validity preview effect	10.17	5.72	1.78
Go-past duration (ms)	Intercept	**317.3**	**12.16**	**26.1**
	Orthographic preview effect	− **16.85**	**7.4**	− **2.28**
	Syntactic validity preview effect	5.9	7.35	0.8
Regression out (%)	Intercept	**1.54**	**0.12**	**13.3**
	Orthographic preview effect	0.1	0.1	1.14
	Syntactic validity preview effect	0.01	0.1	0.1

Significant effects are indicated in bold

**Table 6:** Results of the (generalized) linear mixed-effects models for fixation duration and probability measures for the noun in Experiment 2

Measure	Fixed effect	*b*	SE	*t/z*
Fixation probability (%)	Intercept	**2.55**	**0.18**	**14.02**
	Orthographic preview effect	**− 0.33**	**0.01**	**− 3.74**
	Syntactic validity preview effect	0.07	0.1	0.46
First fixation duration (ms)	Intercept	**232.22**	**4.49**	**51.75**
	Orthographic preview effect	**− 11.52**	**2.38**	**− 4.85**
	Syntactic validity preview effect	**5.67**	**2.17**	**2.61**
Gaze duration (ms)	Intercept	**258.61**	**6.52**	**39.65**
	Orthographic preview effect	**− 18.03**	**3.14**	**− 5.74**
	Syntactic validity preview effect	**9.57**	**3.41**	**2.81**
Go-past duration (ms)	Intercept	**378.89**	**15.99**	**23.7**
	Orthographic preview effect	**− 40.49**	**9.04**	**− 4.48**
	Syntactic validity preview effect	**28.85**	**9.85**	**2.93**
Regression out (%)	Intercept	**1.2**	**0.21**	**5.61**
	Orthographic preview effect	**0.21**	**0.08**	**2.74**
	Syntactic validity preview effect	**− 0.18**	**0.07**	**− 2.52**

Significant effects are indicated in bold

#### Article viewing times

Readers were equally likely to fixate the article in the identical, syntactic matching, and syntactic mismatching preview condition [both |*z*|s < 1]. There were significant differences between the identical and the syntactically matching preview condition with shorter first fixation duration on the article after an identical preview [*t* = − 2.3]. The go-past time was also increased when a syntactically matching compared to an identical preview was presented [*t* = − 2.28].^1^ There was no difference between the two conditions regarding gaze duration [*t* = − 1.64]. Likewise, the probability for an outgoing regression was not affected by a syntactically matching in comparison with an identical mask [*z* = 1.14].

**Fig. 6:**
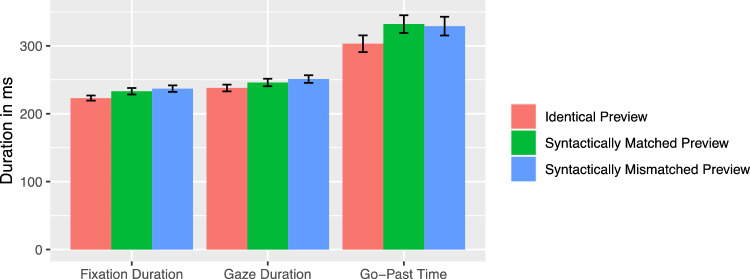
Viewing times on the article as a function of preview condition (Experiment 2). Error bars represent ± 1 standard error of the mean, calculated within-subjects

The first fixation duration, gaze duration, and go-past time showed no syntactic validity preview effect [all |*t*|s < 1.8, see Fig. 6]. Similarly, the effect did not show up in different probabilities for an outgoing regression [*z* = 0.1].

#### Noun viewing times

The probability that the noun was fixated was higher in the syntactically matching mask condition than in the identical mask condition [*z* = − 3.74]. But readers were equally likely to fixate the noun in the syntactically matching and mismatching condition [*z* = 0.75].

Across all reading measures, there was a significant difference between the identical and the syntactically matching preview condition. The viewing durations were longer when a syntactically matching noun preview was presented compared to an identical preview [all |*t*|s 0 > 4.48]. In addition, a greater proportion of outgoing saccades were progressive after an identical preview was presented compared to the syntactically matched preview [*z* = 2.74].

**Fig. 7:**
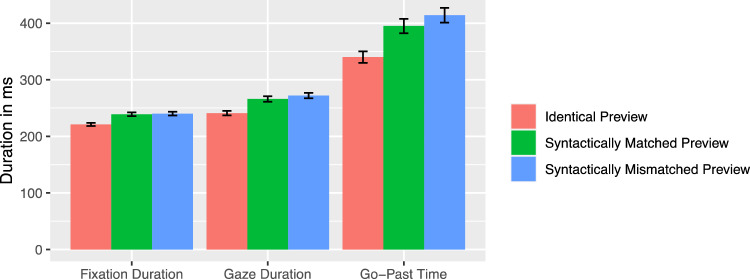
Viewing times on the noun as a function of preview condition (Experiment 2). Error bars represent ± 1 standard error of the mean, calculated within-subjects

There was a significant preview effect of the syntactic match across all viewing time measures. The first fixation duration, gaze duration, and go-past duration were increased when a syntactically mismatched preview was presented compared to a syntactically matching preview [all |*t*|s > 2.61, see Fig.7]. This effect was also evident in a higher number of regressions out of the noun [*z* = − 2.52] when the preview was syntactically mismatching.

**Fig. 8:**
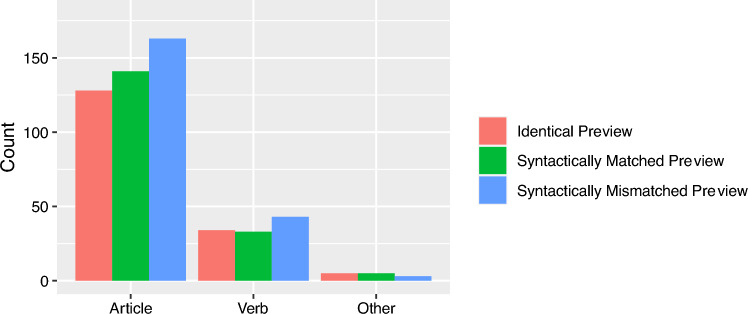
Regressions out of the noun as a function of preview condition and target word (Experiment 1)

Further analysis of the regression target revealed that with 77.84% most of the regressions landed on the article, 19.81% on the verb, and 2.34% on other words at the beginning of the sentences (see Fig. 8  for absolute values). Numerically, the article was fixated again especially for syntactically inappropriate masks. However, there were no significant differences for the regression target between the identical and syntactic matching preview [*b* = 0.1, SE = 0.16, *z* = 0.66] and the syntactic matching compared to the mismatching preview [*b* = − 0.5, SE = 0.15, *z* = − 0.34].

### Discussion

In Experiment 2, as in Experiment 1, subjects read sentences for comprehension in which the syntactic fit of a nominal phrase was manipulated. This time, however, the noun and not the article was varied. There were three conditions in which the noun was presented, either identically, syntactically matching or syntactically mismatching in the parafovea. In this design, effects of orthographic and syntactic mismatch could be dissociated. The results clearly indicated that the effect of a combined manipulation on both levels was substantially larger compared to the isolated orthographic manipulation. This provided solid evidence for the utilization of extrafoveal syntactic information during reading in German. When the article was fixated, the orthographic mismatch in both preview conditions led to an increase in fixation durations. For fixations on the noun, effects on the orthographic level were evident in all reading measures, as could be expected from any disruption of basic word processing. More interesting was the fact that the additional syntactic effect was also evident in all reading parameters except fixation probability. The benefit from a gender matching preview relative to a gender mismatching preview was apparent in both shorter viewing times and less outgoing regressions.

These results confirm that syntactic information with regard to grammatical gender is accessible at an early stage of processing, when the source if the critical information is still located in the parafovea. The increased processing load is carried to later stages, and the increased frequency of regressions to words earlier in the sentence suggests that the readers attempt to deal with the perceived syntactic incongruity.

## General discussion

The main aim of this study was to illuminate the spatially distributed processing of grammatical gender information during the reading of sentences in German. Previous research had shown that various factors can influence parafoveal word processing. There is evidence that orthography, phonology, morphology, and, to some extent, semantics can be processed in the parafovea (see Schotter et al., 2012, for a detailed discussion). Evidence on parafoveal preprocessing of the subsequent word (N + 1) is quite robust after several decades of research. To what extent these effects can be extended to the next word (N + 2) is the subject of ongoing debate (e.g., Vasilev & Angele, 2017).

So far, relatively little attention has been paid in the eye-tracking literature to the time line of syntax processing during sentence reading. Initial studies pointed to the possibility that syntax in the form of word type may be processed early, with effects even on the probability of fixating a parafoveally visible word (Brothers & Traxler, 2016; Veldre & Andrews, 2018). To our knowledge, syntax in the form of grammatical gender has not been part of eye movement research so far. The results of the present study, therefore, add a new angle to the debate, suggesting that grammatical gender can have a substantial role in online reading comprehension and may influence early stages of processing.

In our present work, evidence for parafoveal processing of gender-related syntactic information was present in both experiments. In Experiment 1, these effects materialized both for fixations on the article and the noun. The article had a higher probability of being fixated and the gaze duration and go-past time were increased when a syntactical mismatch had been presented. For the noun, both experiments show a strong effect of syntactic fit, which is reflected in all viewing time measures. These effects are most pronounced in late processing and regressions occur more frequently with syntactically mismatched parafoveal masks.

Before looking further into the central issue of parafoveal syntax processing, we will first consider the fact that our study adds to the accumulating body of literature on orthographic preview effect for word N + 1 (Experiment 1) and, more importantly, word N + 2 (Experiment 2). Our findings are clearly in line with earlier evidence on N + 2 preview effects, especially for a short word N + 1 (see also Kliegl, et al., 2007; Angele & Rayner, 2011; Radach et al., 2013; Cutter et al., 2017; Risse & Kliegl, 2012).

Taking the orthographic preview effect as a baseline, our Experiment 2 provides the first direct evidence that syntactic (gender) information can be extracted from a parafoveal word N + 2 during normal reading. Both parafoveal nouns, the one visible before and the one visible after the display change, were equally appropriate in the general context of the sentence. It is therefore the joint processing (either parallel or sequential) of both article and noun that must have created the viewing time effects we observed. Considering the dynamics of sentence reading, the article may provide a cue to the gender of the subsequent noun, serving as a constraint in the activation of word candidates. Ideally, suitable lexical candidates would be limited to syntactically fitting nouns, either in terms of facilitation for matching candidates or inhibition for non-matching words (see Balota et al., 1985, for a seminal discussion on how parafoveal processing and contextual constraint may interact during sentence reading).

Looking at this problem from the viewpoint of the E–Z reader model (Reichle et al., 1998), our results appear problematic on two levels. First, within the eye movement control machinery of this model, N + 2 preview effects should usually not occur on the lexical level. More specifically, the extraction of linguistic information from word N + 2 during the fixation of word N would require several steps: first, the completion of the L2 processing of word N, then, the completion of L1 and L2 processing of word N + 1, and finally, some amount of L1 processing of word N + 2. For this to happen, the L2 processing of word N and the full recognition of N + 1 would have to be exceedingly efficient (Radach et al., 2013). This assumption appears not implausible for our sentence materials, as the articles serving as N + 1 words are short and extremely familiar. Indeed, Rayner et al. (2007) argued that N + 2 preview effects may be possible when N + 1 words are short and common. Under such favorable linguistic processing conditions, the time line of L1 and L2 processing can approximate zero in serial attention shift models, and processing conditions might be established within which N + 2 preview effects can be accommodated.

However, evidence for N + 2 effects on the level of syntactic processing adds another layer of difficulty for sequential processing accounts. Staub (2011) performed a series of simulations using the E–Z Reader 10 model. He tested, among other aspects, whether and under which conditions post-lexical integration difficulties can have an influence on fixation probability. Similarly, Schotter and Leinenger (2012) discussed ways to accommodate higher-level parafoveal preview effects within a serial processing framework that preserves the assumption that such effects are due to failures in post-lexical integration.

We believe that this assumption appears hard to reconcile with our data. In our Experiment 1, the article was skipped more often when there was no syntactic fit with the noun. The decision to send a saccade to the article vs. the noun must be made no later than 70 ms before the end of the current fixation on word N (Deubel et al., 2000). Acquisition and utilization of syntactic information from word N + 2 to an extent that modifies the targeting decision for the next saccade at this early point (about 150 ms into an average fixation on word N) appear quite incompatible with the idea of post-lexical integration of syntactic information from two adjacent words.

Alternatively, article and noun may be processed simultaneously (at least with substantial temporal overlap), so that letter information accumulates in parallel from both words within the perceptual span (e.g., Inhoff et al., 2006) Reilly & Radach, 2006; Snell et al., 2018). The syntactic relationship of the two words would be gradually processed, as the transition from visual to orthographic and lexical information proceeds. Similarly, Snell et al. (2017) proposed that multiple words can be processed in parallel beyond the sub-lexical level, leading to higher order integration of information from several words’ positions within the current perceptual span.

Within such a parallel processing scenario, article and noun might be processed as one functional unit, initially forming a joint perceptual entity and subsequently a cognitive unit of meaning. The possibility that noun phrases may be processed in terms of an integrated “word group” has been initially proposed by Radach (1996); see Drieghe et al. (2008) for more recent empirical work and a detailed discussion. As the combination of articles and nouns in German is largely arbitrary (despite heroic attempts of teachers to provide rules, e.g., Vayenas, 2017), it appears possible that such two-word units are often explicitly represented in the mental lexicon, allowing for fast processing as a quasi-lexical unit. At this point, this idea has to remain speculative and open for subsequent research.

The idea that frequent minimal noun phases are processed as in integrated functional unit also offers an answer to the seemingly puzzling question why gender-specific articles matter at all in written German. Indeed, if all articles would be the same, as it is the case in English, or if all articles were omitted, German text would look and sound quite awkward, but it could still be read without any major loss in the understanding of sentence meaning. Apparently, the processing system takes the language structure as it is and utilizes each source of information within the perceptual span pragmatically for an optimal flow of information acquisition (see, e.g., Schmuck, 2020, for a linguistic treatise on the grammar of definite articles in German, Dutch and English).

In addition to the effects of parafoveal syntactic information on early oculomotor measures such as fixation probability for the two elements within the noun phrase, we also found substantial effects on late measures up to an increased frequency of regressive saccades (see Inhoff et al., 2019, for a recent review). The mismatch of gender information perceived in the parafovea leads to allocation of substantial resources into attempts to reconcile the seemingly conflicting syntactic information. As the German language allows a very flexible sentence structure, part of this re-analyses could be a check on whether the article actually refers to the noun. In addition, the grammatical gender of the word can be flexible due the productive nature of compounding nouns in German (Inhoff et al., 2000). If these potential problems were the main issue in re-analysis, regression should go to locations to the left of the noun phrase. This is clearly not the case, as in both experiments, most regressions target the article. The large number of regressions landing on the article is particularly striking in Experiment 2, where the noun was changed in the parafovea. The fact that immediate re-analyses is so strongly localized within the noun phrase underscores its importance as an integrated functional unit.

Our results are in harmony with a series of gender priming experiments using EEG methodology in German, indicating that grammatical gender can be processed early during the time-course of linguistic processing (Friederici & Jacobsen, 1999). This conclusion is also reinforced by results of a further ERP work where a syntactic mismatch was found to be associated with the occurrence of an early left anterior negativity (ELAN; Deutsch & Bentin, 2001; Gunter et al., 2000). In these experiments, however, there was always an overt violation of syntax, which could have led to expectancy effects and specific strategies. In contrast, our work demonstrated early effects of syntactic processing in an ecologically valid, natural reading situation.

In conclusion, the presents work contributes to the growing evidence in favor of spatially distributed processing of syntactic information in different written alphabetic languages, such as English (Brothers & Traxler, 2016; Veldre & Andrews, 2018), Dutch (Snell et al., 2017), and now German. Converging results have also been obtained for Korean, a non-alphabetic, predominantly syllabic writing system, by Kim et al. (2012). These authors reported that readers of Korean parafoveally process specific characters located at the rightmost position within a word, which serve to indicate the syntactic class (and semantic role) of nouns within a sentence.

The fact that in these studies evidence for parafoveal processing of syntactic information has emerged in early oculomotor measures provides evidence in favor of immediate and interactive rather than late and modular linguistic processing during reading (see Tanenhaus & Lucas, 1987, for a detailed account of the modular view). It has recently been argued quite convincingly, that there is still good evidence for modularity during sentence processing (Ferreira & Nye, 2018), so that it may to some extent depend on the particular construction and local processing constraints whether early distributed syntactic processing can indeed take place.

## Data Availability

A permanent version of our data, materials, and analysis code for both experiments can be found at https://osf.io/rh4p5.

## Appendix 1

Sentence material from Experiment 1. Within the sentences, the syntactic fit of the nominal phrase and the frequency of the noun were varied. Each subject was presented with only one of the four versions (matching/mismatching Article; high/low frequent noun).

1. Ausgerechnet am Montagmorgen fällt die/der Tasse/Schale zu Boden und zerbricht in viele Teile. [Just on Monday morning, the cup/bowl falls to the ground and breaks into many pieces.]
2. Den Mitbewohner stört das/die Blech/Chaos in der Küche, da es das Kochen unnötig erschwert. [The roommate is bothered by the tray/chaos in the kitchen as it makes cooking unnecessarily difficult.]
3. Auf der bergigen Insel liegt der/das Schatz/Orden schon seit hunderten Jahren vergraben. [On the mountainous island the treasure/medal has been buried for hundreds of years.]
4. Schon seit seiner Kindheit liebt der/die Bauer/Hirte es, von vielen Tieren umgeben zu sein. [Since childhood, the farmer/shepherd has loved being surrounded by many animals.]
5. Der ausgebrochene Verfolgte sieht den/das Spion/Späher und kann ihm gerade noch entkommen. [The escaped fugitive sees the spy/scout and manages to escape just in time.]
6. Durch die Planänderungen passt das/der Kostüm/Gewand nicht mehr zum neuen Motto der Feier. [Due to the changes in plan, the costume/garment no longer fits the new theme of the celebration.]
7. Der psychisch Kranke kennt den/das Teufel/Satan persönlich, erzählt er seinem Therapeuten. [The mentally ill person knows the devil/Satan personally he tells his therapist.]
8. Wegen der streikenden Müllabfuhr liegt der/die Dreck/Abfall schon viel zu lange vor dem Haus. [Due to the striking garbage disposal, the dirt/waste has been lying in front of the house for far too long.]
9. Der geduldige Vater zieht den/die Fisch/Hecht mit einigem Schwung aus dem tiefen Wasser. [The patient father pulls the fish/pike out of the deep water with some force.]
10. In dem schönen Kindermärchen sitzt der/das Drache/Troll im verwunschenen Wald an einem Bach. [In the beautiful children's fairy tale, the dragon/troll sits by a stream in the enchanted forest.]
11. Nach dem Besuch beim Tierarzt kriegt das/der Pferd/Fohlen zur Belohnung eine dicke Mohrrübe. [After visiting the vet, the horse/foal gets a big carrot as a reward.]
12. Das kleine Mädchen suchte den/das Kasten/Sender mit der lustigen Einspielmusik ohne Erfolg. [The little girl searches for the box/transmitter with the funny intro music unsuccessfully.]
13. Zum wiederholten Male schon setzte der/die Idiot/Tölpel sich auf die frisch gestrichene Bank. [For the umpteenth time, the idiot/booby sits on the freshly painted bench.]
14. Zum Entsetzen der Schulklasse fliegt der/die Vogel/Geier ganz knapp über ihre Köpfe hinweg. [To the horror of the school class, the bird/vulture flies just over their heads.]
15. Schon in den frühen Morgenstunden trifft der/das Agent/Makler sich mit seinem neuen Klienten. [Early in the morning, the agent/realtor meets his new client.]
16. Dem frisch verheirateten Paar gefiel die/der Reise/Safari in den afrikanischen Nationalpark. [The newly married couple enjoyed the trip/safari to the African national park.]
17. Meine liebe Tante suchte den/die Knopf/Faden der selbst genähten Hose farblich passend aus. [My dear aunt chooses the button/thread for her self-sewn pants to match the color.]
18. Die beunruhigte Mutter lässt den/das Kummer/Groll der letzten Tage hinter sich und entspannt. [The worried mother leaves the sorrow/resentment of the past few days behind and relaxes.]
19. Die neue Mitarbeiterin zeigt das/der Zimmer/Resort zu Beginn und verweist auf die Hausregeln. [The new employee shows the room/resort at the beginning and refers to the house rules.]
20. Bei der wöchentlichen Kaffeerunde redet die/der Mutter/Uroma über ihren Besuch in der Altstadt. [During the weekly coffee round, the mother/great-grandmother talks about her visit to the old town.]
21. Der Leiter der Einrichtung sieht die/das Hilfe/Pflege durch Angehörige als förderlich an. [The head of the facility sees the help/care provided by relatives as beneficial.]
22. Viele ältere Reisende können die/der Hitze/Wärme in den kleinen Hotelzimmern nicht ertragen. [Many older travelers cannot bear the heat/warmth in the small hotel rooms.]
23. Der erschöpfte Bauer fährt den/die Wagen/Karren nach der anstrengenden Arbeit in die Scheune. [The exhausted farmer drives the car/cart into the barn after the strenuous work.]
24. Für einen jungen Lehrling nimmt die/der Übung/Praxis einen großen Teil der Ausbildung ein. [For a young apprentice, practice/practice takes up a large part of their training.]
25. Nach dem üppigen Mahl bleibt der/das König/Herzog noch bei Tisch und unterhält sich ausgiebig. [After the sumptuous meal, the king/duke stays at the table and talks extensively.]
26. Die hübsche Bäckerin liebt den/das Bauern/Müller schon seitdem beide kleine Kinder waren. [The pretty baker has loved the farmer/miller since they were both small children.]
27. Zur Freude der Eltern stand die/der Lampe/Kerze nun auf dem neuen Nachttisch neben dem Bett. [To the joy of the parents, the lamp/candle is now on the new nightstand next to the bed.]
28. Nach dem leckeren Mittagessen bleibt der/die Junge/Bengel noch einige Zeit am Tisch sitzen. [After the delicious lunch, the boy/rascal stays at the table for a while.]
29. Zur Freude der Söhne konnte der/die Streit/Disput auf friedliche Weise ausgetragen werden. [To the delight of their sons, the dispute/disagreement was resolved peacefully.]
30. Für jeden neuen Gefangenen sieht der/das Knast/Bunker zu Beginn sehr Furcht einflößend aus. [To every new prisoner the prison/bunker looks very intimidating at the beginning.]
31. Die Entwicklung von Shopping-Apps bringt den/die Handel/Tausch von Kleidung enorm voran. [The development of shopping apps is greatly advancing the trade/exchange of clothing.]
32. Der gestresste, müde Student packt die/der Butter/Gurke hektisch in seine Einkaufstasche. [The stressed, tired student hurriedly puts the butter/cucumber into their shopping bag.]
33. Ein romantisches Liebeslied singt der/die Bruder/Sänger der Braut am Tag ihrer Hochzeit. [A romantic love song sings the brother/singer of the bride on her wedding day.]
34. Bei der Untersuchung fühlt der/das Doktor/Helfer vorsichtig, ob die Knochen verheilt sind. [During the examination, the doctor/assistant gently feels to see if the bones have healed.]
35. Der junge Student möchte die/der Krise/Armut überwinden und engagiert sich ehrenamtlich. [The young student wants to overcome the crisis/poverty and volunteers.]
36. Zur Überraschung der Gäste macht der/die König/Regent eine sehr erfreuliche Ankündigung. [To the surprise of the guests, the king/ruler makes a very pleasing announcement.]
37. Die Verantwortung für die grausame Tat trägt der/das Mörder/Gauner sein ganzes Leben lang. [The responsibility for the cruel deed carries murderer/scoundrel for his entire life.]
38. Wegen der neuen Anlage hörte der/die Alarm/Notruf sich völlig anders als die Tage zuvor an. [Because of the new system, the alarm/emergency call sounded completely different than it did in the previous days.]
39. Den langjährigen Mitarbeiter nervt die/das Sache/Misere mit den neuen Geräten im Betrieb. [The long-term employee is annoyed by the issue/problem with the new equipment in the company.]
40. Bis in die Mittagsstunden liegt der/die Cousin/Vetter in seinem gemütlichen, großen Bett. [Until midday, the cousin/cousin lies in his comfortable, large bed.]
41. Nicht auf den ersten Blick sichtbar steht der/das Koffer/Kübel hinter einem Tisch verborgen. [Not visible at first glance, the suitcase/bucket is hidden behind a table.]
42. Die reichen Käufer prüfen die/der Tiefe/Länge des gefertigten Schrankes erneut sehr genau. [The wealthy buyers recheck the depth/length of the manufactured wardrobe carefully.]
43. Entgegen aller Erwartungen bleibt der/die Besuch/Klient noch bis in die späten Abendstunden. [Contrary to all expectations, the visitor/client stays until late in the evening.]
44. Die erholten Urlauber lieben die/der Fahrt/Etappe entlang des schmalen, natürlichen Baches. [The refreshed vacationers love the journey/leg along the narrow, natural stream.]
45. Der Lagerarbeiter nimmt die/das Liste/Kartei und geht alle Punkte noch einmal genau durch. [The warehouse worker takes the list/file and goes through each item again carefully.]
46. Der flinke Arbeiter wusste das/die System/Gefüge der Balken so zu legen, dass es stabil ist. [The nimble worker knew how to lay the system/structure of the beams so that it was stable.]
47. Laut eines Artikels bringt das/der Spiel/Puzzle Spaß und Spannung für die ganze Familie. [According to an article, the game/puzzle brings fun and excitement for the whole family.]
48. Der mutige Abenteurer liebte die/das Gewalt/Härte der Natur in den verlassenen Gegenden. [The brave adventurer loved the violence/harshness of nature in the deserted areas.]
49. Das tierliebe Mädchen rettet die/der Fliege/Biene aus dem Limonadenglas vor dem Ertrinken. [The animal-loving girl saves the fly/bee from the lemonade glass before it drowns.]
50. Der kleine Junge lässt die/das Katze/Ziege auf dem Bauernhof in den Wohnbereich der Familie. [The little boy brings the cat/goat into the living area of the family on the farm.]
51. Den Pferden auf der Koppel machte der/die Krach/Donner des aufziehenden Gewitters nichts aus. [The horses in the paddock were not bothered by the noise/thunder of the approaching storm.]
52. Der frustrierte Mann bringt den/die Tisch/Beamer zurück in den Laden, um ihn zu reklamieren. [The frustrated man returns the table/projector to the store to claim it.]
53. Der innovative Künstler stellt das/der Stück/Glied des großen Werkes im Schaufenster aus. [The innovative artist displays the piece/part of the large artwork in the shop window.]
54. Auf den Betrachter wirkt der/die Schein/Glanz des gerade enthüllten Kunstwerkes surreal. [To the viewer appears the shine/glow of the just unveiled artwork appears surreal.]
55. Im geheimnisvollen Keller steht die/der Kiste/Truhe und darf von niemandem geöffnet werden. [In the mysterious cellar is the chest/trunk and must not be opened by anyone.]
56. Vor dem Versand zum Kunden werden die/der Farbe/Nuance und Qualität der neuen Ware geprüft. [Before shipping to the customer, the color/shade and quality of the new goods are checked.]
57. Der glückliche Mann kriegt die/das Reise/Jacht geschenkt und freut sich über den Hauptgewinn. [The lucky man is given the trip/yacht as a gift and is delighted with the grand prize.]
58. Die Taxifahrerin möchte den/die Dienst/Dialog so schnell wie möglich hinter sich bringen. [The taxi driver wants to get the service/dialogue over with as quickly as possible.]
59. Die erschöpften Studenten holen das/der Essen/Sushi im beliebten Restaurant an der Ecke ab. [The exhausted students pick up the food/sushi at the popular restaurant on the corner.]
60. Mit großer Neugierde liest der/die Autor/Texter die vor kürzlich erschienenen Werke der Kollegen. [With great curiosity, the author/writer reads the recently published works of colleagues.]
61. Der sensiblen Schauspielerin macht die/der Presse/Kritik schon immer nervlich zu schaffen. [The sensitive actress has always been bothered by the press/criticism.]
62. Das kleine Mädchen kannte den/die Fluss/Teich sehr gut, da sie im Sommer oft dort spielte. [The little girl knew the river/pond very well, as she often played there in the summer.]
63. Im Heimatort und dem Umland fällt die/das Gruppe/Clique wegen krimineller Machenschaften auf. [In the hometown and surrounding area, the group/clique is known for their criminal activities.]
64. In den dämmrigen Abendstunden sehen die/der Augen/Ordner nur undeutliche Schemen und Schatten. [In the dim evening hours, the eyes/stewards only see indistinct shapes and shadows.]
65. Die gewissenhafte Sekretärin bringt den/die Brief/Beleg umgehend zu ihrem Chef ins Büro. [The conscientious secretary immediately brings the letter/receipt to her boss's office.]
66. Je nach Schweregrad nimmt die/der Menge/Dosis des zu verabreichenden Medikaments zu oder ab. [Depending on the severity, the amount/dose of the administered medication increases or decreases.]
67. Für die Bewohner des Dorfes sorgt der/die Sturm/Orkan für eine lebensbedrohliche Notlage. [For the village residents, the storm/hurricane poses a life-threatening emergency.]
68. Wie kein Anderer konnte der/das Leiter/Mentor die Motivation seiner Mitarbeiter fördern. [Like no one else, the leader/mentor was able to promote the motivation of his employees.]
69. Die gesättigte Frau zahlt die/der Pizza/Pasta und verlässt das hübsche, gute Restaurant. [The satiated woman pays for the pizza/pasta and leaves the pretty, good restaurant.]
70. Nach der Diskussion letzten Jahres stellt die/das Truppe/Clique sich den Fragen der Bewohner. [After last year's discussion, the group/clique answers the residents' questions.]
71. Die verzweifelten Wanderer sehen die/der Lampe/Fackel als letzte Rettung im dunklen Wald. [The desperate hikers see the lamp/torch as their last resort in the dark forest.]
72. Alle Bewohner des Hauses sahen das/die Schild/Plakat mit den grellen Farben an der Einfahrt. [All the residents of the house looked at the sign/poster with the bright colors at the entrance.]
73. Die Jugendlichen gehen die/das Straße/Gosse entlang und grölen dabei ausgelassen im Chor. [The teenagers walk along the street/gutter and cheer boisterously in unison.]
74. In schwierigen Situationen hilft das/die Gefühl/Gespür für die richtige Entscheidung weiter. [In difficult situations, the feeling/sense for the right decision helps.]
75. Die kreative Rentnerin konnte das/der Metall/Eisen zu wunderschönen Schmuckstücken formen. [The creative retiree was able to shape the metal/iron into beautiful jewelry pieces.]
76. Wegen der vielen schlechten Noten musste die/das Schar/Klasse den Vokabeltest wiederholen. [Because of the many bad grades, the class/crowd had to repeat the vocabulary test.]
77. Bei drohender Gefahr bietet der/die Höhle/Grube ein sicheres Versteck für kleine Tiere. [In case of imminent danger, the cave/pit offers a safe refuge for small animals.]
78. Die junge Hausfrau liebt den/die Handel/Kiosk am Ende der breiten Einkaufsstraße besonders. [The young housewife especially loves the shop/kiosk at the end of the wide shopping street.]
79. Die Praktikanten müssen den/das Fehler/Mangel am gefertigten Produkt umgehend beseitigen. [The interns must promptly eliminate the error/defect in the manufactured product.]
80. Mancher junge Mensch liebt die/der Chance/Option in einem anderen Land leben zu können. [Some young people love the chance/opportunity to be able to live in another country.]
81. Jeden Samstag fährt der/die Pater/Pastor zum Altersheim, um mit den Senioren zu sprechen. [Every Saturday, the father/priest visits the nursing home to talk to the seniors.]
82. Die modebewusste Frau trägt die/der Schuhe/Bluse zu besonderen Anlässen als Glücksbringer. [The fashion-conscious woman wears the shoes/blouse as a lucky charm for special occasions.]
83. Der erfahrene Soldat kennt die/das Angst/Scheu vor dem nächsten Kampfeinsatz mehr als gut. [The experienced soldier knows the fear/trepidation before the next combat mission more than well.]
84. Die junge Polizistin möchte die/der Waffe/Montur säubern und erledigt dies vor Dienstbeginn. [The young policewoman wants to clean the gun/equipment and does this before starting duty.]
85. Die Hochschwangere bringt das/die Drama/Fiasko so sehr zum Weinen, dass sie laut schluchzt. [The heavily pregnant woman is so moved by the drama/fiasco that she cries loudly.]
86. Nach der Rückkehr aus dem Krieg sagte der/das Freund/Senior kein einziges gutes Wort mehr. [After returning from the war, the friend/senior did not say a single good word anymore.]
87. Die ältere Dame kaufte die/der Blume/Speise an einem kleineren Stand auf dem Wochenmarkt. [The elderly lady bought the flower/dish at a smaller stand at the weekly market.]
88. Zur Freude des Mannes bleibt der/die Mantel/Umhang beim Streichen der neuen Küche unversehrt. [To the man's delight, the coat/cloak remains undamaged while painting the new kitchen.]
89. Für den Erfinder hatte die/der Vision/Utopie eines fliegenden Taxis eine große Bedeutung. [For the inventor, the vision/utopia of a flying taxi had great significance.]
90. Zur Freude der Veranstalter sorgt die/der Aktion/Revue deutschlandweit für großes Aufsehen. [To the organizers' delight, the action/show causes a sensation throughout Germany.]
91. Die angeschlagene Patientin dachte den/die Virus/Tumor bereits erfolgreich bekämpft zu haben. [The sick patient thought they had successfully fought off the virus/tumor already.]
92. Vor jeder Nachtwache spielt der/die Posten/Hüter der Burg mit dem Freund eine Partie Schach. [Before every night watch, the post/guard of the castle plays a game of chess with his friend.]
93. Beim Durchsehen der hölzernen Figuren fehlt der/das Bauer/Läufer des alten Schachspiels. [When going through the wooden pieces, the pawn/bishop of the old chess game is missing.]
94. Der schlauen Studentin gefiel der/die Stoff/Inhalt des Seminars aus dem Wahlpflichtmodul. [The smart student liked the material/content of the seminar from the elective module.]
95. Seit einigen Stunden sitzt der/das Freund/Kumpan in dem Versteck, um jemanden zu erschrecken. [For several hours now, the friend/companion has been sitting in the hiding place to scare someone.]
96. Der begabte Künstler konnte die/der Figur/Statue an einen interessierten Kunden verkaufen. [The talented artist was able to sell the figure/statue to an interested customer.]

## Appendix 2

Sentence material from Experiment 2. Within the sentences, syntactic fit was varied. Each subject was presented with only one of the three versions (identical/syntactically matching/syntactically mismatching).

1. Hoffentlich bringt die Woche/Folge/Monat viel Spaß und viele spannende Momente mit sich. [Hopefully brings the week/episode/month lots of fun and exciting moments.]
2. Die junge Frau guckt die Tasche/Kirche/Strand an und kann sich kaum von ihr lösen. [The young woman looks at the bag/church/beach and can hardly tear herself away from it.]
3. Ein heftiger Windstoß trägt die Blüte/Mütze/Vogel über den Gartenzaun des neuen Nachbarn. [A strong gust of wind carries the blossom/hat/bird over the garden fence of the new neighbor.]
4. Der neugierige Gorilla fasst die Wange/Decke/Boden immer wieder mit großem Vergnügen an. [The curious gorilla touches the cheek/blanket/floor repeatedly with great pleasure.]
5. In dem eisig kalten Winter wurde der Bauer/Vogel/Katze aus seinem Zuhause vertrieben. [In the icy cold winter, the farmer/bird/cat was driven out of his home.]
6. Schon seit sehr langer Zeit trägt der Mensch/Balken/Person die schweren Ziegelsteine. [For a very long time, the human/beam/person has been carrying the heavy bricks.]
7. Auf dem Rückweg schläft der Pilot/Agent/Witwe fast ein und gefährdet damit Menschenleben. [On the way back, the pilot/agent/widow almost falls asleep and thereby endangers lives.]
8. Da das Gefäß ein Loch hat, nimmt die Frau die Kanne/Tasse/Eimer zum Gießen der Blumen. [Since the vessel has a hole, the woman takes the pot/cup/bucket to water the flowers.]
9. Auf dem großen Familienfest trinkt der Onkel/Vater/Tante zu viel von dem Früchtepunsch. [At the big family celebration, the uncle/father/aunt drinks too much of the fruit punch.]
10. Im neu angelegten Garten des Hauses ziert die Hecke/Blume/Busch den Eingangsbereich. [In the newly landscaped garden of the house, the hedge/flower/bush adorns the entrance area.]
11. Das flinke kleine Raubtier frisst die Spinne/Fliege/Frosch mit einem schnellen Bissen auf. [The quick little predator eats the spider/fly/frog with a quick bite.]
12. Nach seinen Erfahrungen bleibt der Fisch/Vogel/Katze niemals an der gleichen Stelle. [Based on his experience, the fish/bird/cat never stays in the same place.]
13. Laut Karte sollte der Platz/Markt/Halle nur einige Meter von seinem Hotel entfernt sein. [According to the map, the square/market/hall should only be a few meters away from his hotel.]
14. Für die kleinen Kinder wirkt der Keller/Garten/Kammer mit seinen dunklen Ecken unheimlich. [For the little children, the cellar/garden/chamber with its dark corners seems spooky.]
15. In einem schlimmen Albtraum macht die Falle/Kraft/Feind dem kleinen Jungen sehr Angst. [In a bad nightmare, the trap/power/enemy scares the little boy very much.]
16. Im Heimatmuseum konnte die Waffe/Kiste/Wagen aus dem alten Persien besichtigt werden. [At the local history museum, the weapon/box/carriage from ancient Persia could be viewed.]
17. In das extravagante Wohnzimmer passt die Lampe/Figur/Kamin vom Stil her vorzüglich hinein. [To the extravagant living room the lamp/figure/fireplace fits in perfectly regarding the style.]
18. Vor lauter Erschöpfung fällt der Vogel/Engel/Katze plötzlich kraftlos auf den Boden. [Due to exhaustion, the bird/angel/cat suddenly falls powerless to the ground.]
19. Die Eltern finden die Gewalt/Gefahr/Unsinn im Spielfilm nicht geeignet für ihre Tochter. [The parents find the violence/danger/nonsense in the movie unsuitable for their daughter.]
20. Wegen einer Unvorsichtigkeit sticht der Draht/Nagel/Nadel den Arbeiter in den Finger. [Due to carelessness, the wire/nail/needle pierces the worker's finger.]
21. In dem großen Einzugsgebiet hatte der Anwalt/Doktor/Schule mit Abstand den besten Ruf. [In the large catchment area, the lawyer/doctor/school had by far the best reputation.]
22. Bei einem wissenschaftlichen Experiment führte der Schein/Schall/Stimme zu leichter Panik. [In a scientific experiment, the appearance/sound/voice led to mild panic.]
23. Bei dem großen Sonntagsbuffet ekelt die Masse/Wurst/Speck die empfindliche Kellnerin an. [At the large Sunday buffet, the mass/sausage/bacon disgusts the sensitive waitress.]
24. An kalten Wintertagen fühlt der Nacken/Wirbel/Leiste sich ganz besonders unangenehm an. [On cold winter days, the neck/vertebra/groin feels particularly uncomfortable.]
25. Laut Empfehlung wird der Kaffee/Kuchen/Speise genossen, wenn er noch frisch und heiß ist. [According to recommendation, the coffee/cake/dish is enjoyed when it is still fresh and hot.]
26. Bei der internationalen Konferenz zieht die Kultur/Gruppe/Planet Aufmerksamkeit auf sich. [At the international conference, the culture/group/planet attracts attention.]
27. In der Broschüre steht der Keller/Zugang/Straße als gruseligster Ort der Stadt beschrieben. [In the brochure, the cellar/entrance/street is described as the scariest place in the city.]
28. Im Haushaltswahrengeschäft liegt die Decke/Seide/Stoff im dekorierten Schaufenster. [In the store for household goods, the blanket/silk/fabric is in the decorated shop window.]
29. Das neugierige kleine Kind möchte die Mauer/Stufe/Stuhl auf jeden Fall hinaufklettern. [The curious little child definitely wants to climb up the wall/step/chair.]
30. In der großen Sporthalle bewegt der Mensch/Tänzer/Gruppe regelmäßig Muskeln und Gelenke. [In the large sports hall the human/dancer/group regularly moves muscles and joints.]
31. Trotz aller Sorgfalt hängt der Knopf/Faden/Kette unabsichtlich an ihrem Kragen herab. [Despite all care, the button/thread/chain unintentionally hangs down from her collar.]
32. Direkt hinter ihrem Haus liegt der Hügel/Platz/Mauer und bietet kaum einen Schutz. [Directly behind her house lies the hill/square/wall and offers little protection.]
33. Die Mutter spürt die Freude/Magie/Stolz beim Betrachten des Theaterstücks ihrer Tochter. [The mother feels the joy/magic/pride when watching her daughter's play.]
34. Bei Anbruch der Dunkelheit läuft der Krebs/Fuchs/Ratte schnell in sein Versteck zurück. [At nightfall, the crab/fox/rat quickly runs back to its hiding place.]
35. Der modischen Frau steht der Gürtel/Mantel/Tasche laut der Verkäuferin ausgesprochen gut. [The fashionable woman looks great in the belt/coat/bag according to the saleswoman.]
36. In den Straßen des Dorfes findet die Tante/Waise/Junge sich mittlerweile gut zurecht. [In the streets of the village, the aunt/orphan/boy is now getting along well.]
37. In dem kleinen Teich wirkt der Krebs/Stein/Figur bei hellem Sonnenschein besonders schön. [In the small pond, the crab/stone/figurine looks particularly beautiful in bright sunshine.]
38. In dem Dokument zeigt die Reihe/Linie/Pfeil den Posten an, der bezahlt werden muss. [In the document, the row/line/arrow indicates the post that needs to be paid.]
39. Wegen der unerwarteten Mieterhöhung zieht die Tante/Sippe/Onkel in ein kleineres Haus. [Because of the unexpected rent increase, the aunt/clan/uncle moves to a smaller house.]
40. Für den Künstler passt die Musik/Farbe/Klang perfekt in sein neustes Arrangement. [For the artist the music/color/sound is a perfect fit for his latest arrangement.]
41. Als kleine Überraschung macht der Besuch/Bruder/Mutter am Morgen ein leckeres Sektfrühstück. [As a small surprise, the visitor/brother/mother makes a delicious champagne breakfast in the morning.]
42. Tage vor der Prüfung lässt die Sorge/Angst/Druck den faulen Schüler kaum noch schlafen. [Days before the exam, the worry/fear/pressure hardly lets the lazy student sleep.]
43. Zur Entspannung am Abend spielt die Köchin/Mutter/Anwalt gerne Schach oder löst Rätsel. [To relax in the evening, the cook/mother/lawyer enjoys playing chess or solving puzzles.]
44. Laut der jungen Verkäuferin gehört die Wolle/Seide/Stoff zum typischen Erscheinungsbild. [According to the young saleswoman, the wool/silk/fabric is part of the typical appearance.]
45. In dem spannenden Gruselfilm liegt der König/Mensch/Person nicht mehr in seinem Sarg. [In the exciting horror film, the king/human/person no longer lies in his coffin.]
46. In der alten Ritterburg spielt der Geist/Prinz/Katze nachts in den düsteren Gängen. [In the old knight's castle, the ghost/prince/cat plays in the dark corridors at night.]
47. Eine Ecke weiter liegt die Straße/Schule/Bäcker nur einige Minuten von der Kirche entfernt. [Around the corner is the street/school/bakery just a few minutes from the church.]
48. In ihrem Gespräch nennt der Mensch/Fahrer/Person sie überraschenderweise bei ihrem Namen. [In their conversation, the person/driver mentions her name surprisingly.]
49. Wegen ihres unhöflichen Benehmens trifft der Blick/Brief/Rache das pubertierende Mädchen. [Because of her rude behavior, the gaze/letter/revenge hits the pubescent girl.]
50. Nach der Zirkusaufführung frisst der Adler/Tiger/Ziege gerne Leckerchen zur Belohnung. [After the circus performance, the eagle/tiger/goat likes to eat treats as a reward.]
51. Die neue Mitarbeiterin wählt die Tasche/Kamera/Mantel für ihren ersten Arbeitstag aus. [The new employee chooses the bag/camera/coat for her first day of work.]
52. Auf dem berühmten Gemälde steht der Ochse/Stier/Ziege am Rande des reißenden Flusses. [On the famous painting, the ox/bull/goat stands at the edge of the raging river.]
53. Schon aus weiter Ferne könnte die Bucht/Buche/Hügel mit einem Fernglas gesehen werden. [Already from afar could the bay/beech/hill be seen from afar with a pair of binoculars.]
54. Laut dem frisch vermählten Brautpaar sollte der Moment/Morgen/Stunde nie zu Ende gehen. [According to the newlyweds, the moment/morning/hour should never end.]
55. Die Putzfrau stellt die Tasse/Mappe/Stift des Mitarbeiters in eine Schublade. [The cleaning lady puts the cup/folder/pen of the employee in a drawer.]
56. Beim Tragen fühlt der Stoff/Anzug/Jacke sich angenehm an, da er aus Seide besteht. [When wearing the fabric/suit/jacket it feels pleasant because it is made of silk.]
57. In der Ecke des Zimmers steht der Stuhl/Tisch/Kiste und verdeckt den Fleck an der Wand. [In the corner of the room is the chair/table/box and covers the stain on the wall.]
58. In der dritten Folge der Serie kommt der Feind/König/Rolle das erste Mal vor. [In the third episode of the series the enemy/king/role appears for the first time.]
59. Seit dem letzten Jahr spart die Klinik/Kirche/Verein gezwungenermaßen in vielen Bereichen. [Since last year, the clinic/church/association has been forced to save money in many areas.]
60. Im Winter kann der Vater/Bauer/Ratte kaum seine große Familie mit genug Essen versorgen. [In winter, the father/farmer/rat can hardly provide enough food for his large family.]
61. In der Mittagssonne rekelt die Katze/Ziege/Tiger sich genüsslich und genießt die Hitze. [In the midday sun, the cat/goat/tiger stretches out and enjoys the heat.]
62. Auch unter starker Belastung reißt der Faden/Stoff/Wolle nur in sehr seltenen Fällen. [Even under heavy load, the thread/fabric/wool only breaks in very rare cases.]
63. Nach dem Sommerurlaub riecht der Abfall/Kuchen/Speise in der Wohnung mehr als unangenehm. [After the summer vacation, smells the garbage/cake/food more than unpleasant in the apartment.]
64. Nach Meinung des Hausmeisters müsste die Schule/Klinik/Garten dringend umgestaltet werden. [According to the janitor, the school/hospital/garden urgently needs to be redesigned.]
65. Schon zu Lebzeiten besaß der König/Prinz/Figur zahlreiche Bewunderer und einige Gegner. [Even during his lifetime, the king/prince/character had numerous admirers and some opponents.]
66. Das hübsche Mädchen umgeht die Gasse/Allee/Platz auf dem langen Weg zu ihrem Freund. [The pretty girl avoids the alley/avenue/square on her long way to her boyfriend.]
67. Das junge Mädchen fürchtet die Gefahr/Arbeit/Termin sehr und kann kaum noch einschlafen. [The young girl is very afraid of the danger/work/appointment and can hardly fall asleep anymore.]
68. Die wissbegierige Grundschülerin fasst die Werke/Kugel/Stein voller Bewunderung an. [The inquisitive elementary school girl touches the works/sphere/stone with admiration.]
69. Trotz ihrer Schreie hilft der Bruder/Lehrer/Klasse dem Mädchen nicht, als es geschubst wird. [Despite her screams, the brother/teacher/class does not help the girl when she is pushed.]
70. Im Garten unseres Nachbarn steht die Figur/Lampe/Stuhl mitten auf der großen Terrasse. [In our neighbor's garden, the statue/lamp/chair stands in the middle of the large terrace.]
71. Das junge Paar läuft die Brücke/Treppe/Garten entlang und redet dabei über die Zukunft. [The young couple walks along the bridge/stairs/garden and talks about the future.]
72. Zurzeit kann der König/Junge/Tante kaum aufstehen oder etwas Essbares zu sich nehmen. [Currently, the king/boy/aunt can hardly get up or eat anything.]
73. Kurz vor der Besprechung wirkt der Druck/Ärger/Sorge sich stark auf die Mitarbeiterin aus. [Shortly before the meeting, the pressure/anger/worry strongly affects the employee.]
74. Durch die große Wucht bleibt der Pfeil/Speer/Waffe in dem harten Baumstamm stecken. [Due to the great force, the arrow/spear/weapon gets stuck in the hard tree trunk.]
75. Trotz der Anstrengungen nimmt der Lehrer/Bruder/Mutter einen Teil der Erziehung auf sich. [Despite the efforts, the teacher/brother/mother takes on a part of the education.]
76. Empfindliche Menschen finden die Farbe/Lampe/Boden im Zimmer nur schwer erträglich. [Sensitive people find the color/lamp/floor in the room difficult to bear.]
77. Die freche Hauskatze leckt die Masse/Milch/Honig vom frisch gewischten Fußboden der Küche. [The cheeky house cat licks the mass/milk/honey from the freshly wiped kitchen floor.]
78. Nach einer langen und anstrengenden Nacht stand der Junge/Knabe/Wache früh morgens auf. [After a long and exhausting night, the boy/young man/guard gets up early in the morning.]
79. Der modebewusste Mann möchte die Jacke/Jeans/Anzug in einer Nummer größer probieren. [The fashion-conscious man wants to try the jacket/jeans/suit in one size larger.]
80. Die hübsche junge Frau kennt die Nummer/Stimme/Besuch nicht und erschrickt deswegen. [The pretty young woman does not recognize the number/voice/visitor and is frightened because of it.]
81. In dem aktuellen Vertrag steht die Summe/Miete/Preis für die Immobilie verbindlich fest. [The current contract stipulates the sum/rent/price for the property as binding.]
82. Für den Betrachter gehört die Ebene/Figur/Bauch zum schönen einheitlichen Gesamtbild. [For the observer, the plain/figure/stomach belongs to the beautiful uniform overall picture.]
83. Der abenteuerliche Junge spürt die Kraft/Sonne/Regen bei der Wanderung in den Alpen. [The adventurous boy feels the power/sun/rain during the hike in the Alps.]
84. Der Verkäufer sucht die Brille/Tasche/Mantel und wird ganz hinten im Lagerraum fündig. [The salesman is looking for the glasses/bag/coat and finds them all the way in the back of the warehouse.]

## Footnotes

1 The difference between the syntactically matching and syntactically mismatching preview was not significant in the analysis of log fixation duration [b = -0.02, SE = 0.01, t = -1.67] and the analysis of log go-past time [b = -0.03, SE = 0.02, t = -1.93]. No other effects changed significance in the analyses of log-transformed data.
